# An Anti-Jamming Method against Interrupted Sampling Repeater Jamming Based on Compressed Sensing

**DOI:** 10.3390/s22062239

**Published:** 2022-03-14

**Authors:** Yingxi Liu, Qun Zhang, Zhidong Liu, Guangming Li, Shichao Xiong, Ying Luo

**Affiliations:** 1 The Institute of Information and Navigation, Air Force Engineering University, Xi’an 710077, China; oliveliuyingxi@sina.com (Y.L.); liuzd13@tsinghua.org.cn (Z.L.); 18792587381@163.com (G.L.); xiongshichao1220@163.com (S.X.); luoying2002521@163.com (Y.L.); 2 The Collaborative Innovation Center of Information Sensing and Understanding, Xi’an 710077, China; 3 The Key Laboratory for Information Science of Electromagnetic Waves (Ministry of Education), Fudan University, Shanghai 200433, China

**Keywords:** inverse synthetic aperture radar (ISAR), interrupted sampling repeater jamming (ISRJ), compressed sensing (CS), anti-jamming

## Abstract

Interrupted sampling repeater jamming (ISRJ) is an attracted coherent jamming method to inverse synthetic aperture radar (ISAR) in the past decades. By means of different jamming parameters settings, realistic dense false targets can be formed around the true target. This paper proposed an adaptive anti-jamming method against ISRJ by adjusting the number of measurements based on compressed sensing (CS). The jamming signal is energy concentrated and segmented sparse in the frequency domain. The measurements number of the reconstructed target signal and the jamming signal is different. According to the restricted isometry property (RIP) condition of CS theory, signal reconstructing performance depends on the number of measurements that varies with the sparsity of the vector. Thus, the jamming signal is suppressed, and the true target signal is retained by altering the measurements number of echo signals. Besides, the two-dimensional (2D) anti-jamming method is derived in detail. The anti-jamming effect is analyzed with different signal-to-noise ratios (SNR), sampling rates, and jam-to-signal ratios (JSR). Simulations prove the effectiveness of the proposed anti-jamming method.

## 1. Introduction

With the increasing types and quantities of targets near space, including aircraft, ballistic missiles, decoys, satellites, near space target, and space debris, inverse synthetic aperture radar (ISAR) plays an increasingly important role in target imaging and identification [1,2]. At the same time, the electronic countermeasures (ECM) technology against ISAR is developing so rapidly with the development of digital radio frequency memory (DRFM). In particular, the interrupted sampling repeater jamming (ISRJ) method based on DRFM can realize deceiving the victim radar by generating amounts of realistic false targets [3,4]. The response speed of ISRJ is fast, and the jamming signal is correlated with the target signal [5,6,7]. Therefore, extensive research of ISRJ has been carried out due to its significant application in electronic warfare.

ISRJ is a novel jamming method against ISAR by interrupting sampling the target echo signal and retransmitting the intercepted segments. The improved ISRJ inherits the traditional “storage-repeater” model and adjusts the sampling interval and retransmission period to conquer the deficiencies of the amplitude decaying rapidly and main false target hysteresis [8,9,10]. Besides, the sub-Nyquist sampling technology is another kind of improved ISRJ method whose sampling rate of the intercepted target echo signal is less than the Nyquist sampling rate [11]. The false targets are induced along with the different orientations, respectively, by interrupted sampling the echo signal along with the corresponding different time domain at sub-Nyquist sampling rate in [12,13,14]. In [14], multiple false targets will be induced along with the range orientation while the CS sampling number should satisfy inequation condition with sparsity. By introducing the sub-Nyquist sampling and transforming the convolution into a parallel formation, the structure for generating the jamming signal is simplified, and the computation complexity is reduced in [7]. The jamming methods mentioned above are mainly focused on one-dimensional jamming. In the field of radar target feature extraction, ISAR imaging using a broadband signal will still be interfered by the ISRJ signal. However, there is a lack of work to study the characteristics of ISRJ jamming signals from the two dimensions of ISAR imaging. Further, this paper investigated the 2D jamming method that the ISRJ imposed both in the fast-time domain and slow-time domain and derived the explicit signal form.

Meanwhile, many anti-jamming methods against ISRJ have attracted much attention. In [15], the jamming signal parameter is estimated firstly, and then an adaptive parameter-adjusted intra-pulse frequency coded signal is transmitted to realize ISRJ suppression. In [16], the jamming signal parameters of intercepted slice number, forwarding times, and slice width was estimated firstly, and then the parameters were used for iterative cancellation of suppression. However, these methods’ performances depend on the precision of parameter estimating. Based on the discontinuous characteristics of the ISRJ signal, [17] proposed a bandpass filter based on the discontinuous pulse duration. A novel method was proposed based on the radar signal energy function, and the results of the function were compared with the threshold to obtain the non-disturbed segments in [18]. According to the energy distribution of the de-chirped signal, a function was constructed to extract the jamming-free signal segments, and the bandpass filter based on these segments was generated in [19]. The filter results are suitable for constant false alarm rate (CFAR) detectors to improve the suppression performance. Nevertheless, the filter method is constructed complicatedly, and it is easily affected by the low signal-to-noise ratios (SNR) or high jam to signal ratio (JSR).

CS theory can reconstruct the sparse data under limited measurements [20,21]. The paper [22] proposed a phase-aided distributed compressive sensing (DCS) method to preserve the target and suppress randomly distributed jamming signals. In [23], a CS-based method for high-resolution ISAR images is proposed under limited measurements. These methods in the aforementioned five papers solve the reconstruction problem based on Compressed Sensing. However, the anti-jamming performance of low SNR and high JSR has not been analyzed.

Since the radar echo signal and ISRJ signal are sparse, the CS method can suppress ISRJ signals [24,25,26]. An imaging method with a non-periodic interrupted sampling-linear frequency modulated signal (NIS-LFM) is proposed in [24]. The NIS-LFM is piecewise sparse, and the target echo can be extracted based on CS theory, while parameters analyses on the anti-jamming performance are limited to restricted isometry property (RIP) conditions. The high-resolution range profile (HRRP) is reconstructed, and this method’s minimum sub-Nyquist sampling rate countering jamming is addressed in [25]. According to the variable sparsity influence, the reconstruction effect, a Bayesian compress sensing-based method is proposed to reconstruct the multiple targets signal by optimizing the sparse model in [26]. This model is based on the unjammed discontinuous segments extracted by the ISRJ strategy already. However, only the range-dimension anti-jamming method is discussed in the above methods, and parameter analysis of CS-based reconstruction is incomplete.

Most of the methods against ISRJ focus on parameter estimation or filter principle, while the anti-jamming method of parameter estimation requires a higher real-time response of hardware. The filter-based anti-jamming method mainly handles the segments without jamming. There is a lack of research on processing directly the radar echo containing jamming signal by means of an algorithm. At present, the available CS-based methods against ISRJ are to filter out the signal without jamming first and then reconstruct the signal with CS theory only using the compressed sensing method to reconstruct the signal. Inspired by the promising CS theory, this paper proposed an adaptive anti-jamming method to suppress the ISRJ signal by adjusting vector sparsity. According to the restricted isometry property (RIP) condition of CS theory, the signal reconstruction effect is relative to the number of the measurements, which varies with the sparsity. In addition, the ISRJ signal is piecewise sparse in the frequency-domain, and the reconstruction sparsity of the target signal is different from that of the ISRJ signal. Hence, the ISRJ signal can be suppressed primarily by searching the number of optimal measurements. This paper first derivates the ISRJ signal model along with range orientation and azimuth orientation and then investigates the 2D anti-jamming method. This method can realize anti-jamming with only a few signal measurements at the receiver and does not need complicated parameter estimation and filter design, which is applicable in various scenarios.

The structure of this paper is organized as follows. In Section 2, a 2D ISRJ signal model is built. In Section 3, the procedure of the 2D anti-jamming method is derived in detail. In Section 4, numerous simulations are carried out. Finally, in Section 5, conclusions are drawn.

## 2. Signal Model

**Figure 1:**
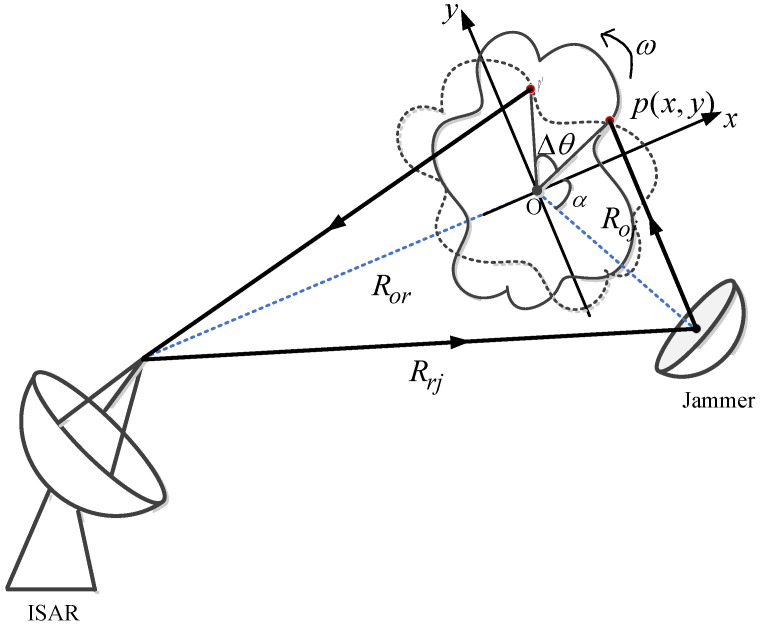
The geometry of the jamming configuration.

The geometry of the jamming configuration is illustrated in Figure 1. The jammer is assumed on the same platform as the radar, and this jamming belongs to main lobe interference. The process of radar receiving jamming signals can be divided into two stages: Firstly, the radar transmits the linear frequency modulation (LFM) wideband waveform, some of which are intermittently intercepted by the jammer and then transmitted to the target. Secondly, the jamming signals are received by the radar receiver, and then multiple false targets are induced in ISAR image. 

In Figure 1, the coordinate *XOY* is established on the point O, which denotes the reference point of the target. The x-axis is the line of the radar transmitting signal. $R_{oj}$, $R_{or}$ and $R_{rj}$ represents the distance between jammer and reference point, radar and reference point, radar, and jammer, respectively. α is the incident angle of the jammer. Assume that the scatter p of the rigid object rotates with speed ω and the instantaneous rotation angle of the scatter p during the observation period is Δθ, which can be expressed as $\omega t_{m}$ with slow time $t_{m}$. Hence, the distance history of ISRJ signal is accumulated after sampling by jammer and reflecting on the scatter p of target, which can be expressed below

(1) $$\begin{array}{c} \left\|R_{p}\right\|=\left\|R_{rj}\right\|+\left\|R_{jp}\right\|+\left\|R_{pr}\right\|\\ \;\;\;\;\;\;\;\;\;\;\;\;=\left\|R_{rj}\right\|+\left\|R_{OJ}\right\|+\left\|R_{Or}\right\|+x\cos\left(\Delta \theta \right)-y\sin\left(\Delta \theta \right)\\ \;\;\;\;\;+x\cos\left(\alpha +\Delta \theta \right)-y\sin\left(\alpha +\Delta \theta \right)\\ \;\;\;\;\;\;\;\;\;\;\;\;\;\;\;\;\;\;\;\;\approx \left\|R_{rj}\right\|+\left\|R_{OJ}\right\|+\left\|R_{Or}\right\|+2\left(x\cos\left(\omega t_{m}+\frac{\alpha }{2}\right)-y\sin\left(\omega t_{m}+\frac{\alpha }{2}\right)\right)\cos\frac{\alpha }{2} \end{array}$$

where x represents the displacement distance of scatter along with the range orientation, while y represents the displacement distance along with the azimuth orientation. The formula transformation of the ISRJ signal during the two stages is described below. Assume that the signal transmitted by radar is as below

(2) $$s(\hat{t},t_{m})=rect(\frac{\hat{t}}{T_{p}})\exp[j2\pi (f_{0}t+\frac{1}{2}\gamma {\hat{t}}^{2})]$$

where $T_{p}$ is the width of the pulse, $f_{0}$ is the carrier frequency and γ is the chirp rate. $\hat{t}$ represents the fast time while $t_{m}$ is the slow time and $t=\hat{t}+t_{m}$ is the full time. The function rect(·) can be described as

(3) $$rect(\frac{\hat{t}}{T_{p}})=\{\begin{array}{cc} 1 & \left|\hat{t}\right|\leq \frac{T_{p}}{2}\\ 0 & \left|\hat{t}\right|>\frac{T_{p}}{2} \end{array}$$

At the jammer, the sampling function in the fast time and in the slow time are, respectively, defined as

(4) $$\begin{array}{l} p\left(\hat{t}\right)=rect(\frac{\hat{t}}{\tau })*\sum_{l_{r}=-\infty }^{+\infty }\delta \left(\hat{t}-l_{r}T_{r}\right)\\ p\left(t_{m}\right)=rect(\frac{t_{m}}{\tau })*\sum_{l_{a}=-\infty }^{+\infty }\delta \left(t_{m}-l_{a}T_{a}\right) \end{array}$$

where τ represents sampling pulse duration and $T_{s}$ is the sampling interval in the fast time. Then the equivalent $D=\tau f_{r}=\frac{\tau }{T_{r}}$ represents the duty ratio, where $f_{r}=\frac{1}{T_{r}}$ is the sampling frequency. $T_{a}$ is the sampling interval in the slow time domain and $f_{a}=\frac{1}{T_{a}}$ is the sampling frequency. This sampling function makes the jamming signal equivalent to the target signal weighted with different Doppler shifts.

The ISRJ signal in the range dimension can be generated by interrupted transmitting the target signal in the fast time domain. The generation processes of ISRJ signal in azimuth orientation are the same as those in range dimension, except that intermittent sampling signal is carried out in the slow time domain with $p\left(t_{m}\right)$. In this section, we take the range dimension as an example. The jamming signal can be expressed as

(5) $$s_{J_{p}}\left(\hat{t},t_{m}\right)=\sigma_{p}\cdot rect\left(\frac{\hat{t}-\frac{R_{p}}{c}}{T_{p}}\right)\cdot \exp\left(j2\pi \left(f_{0}\left(t-\frac{R_{p}}{c}\right)+\frac{\gamma }{2}{\left(\hat{t}-\frac{R_{p}}{c}\right)}^{2}\right)\right)\cdot p\left(\hat{t}\right)$$

where $\sigma_{p}$ is the scattering coefficient of the p point and c is the electromagnetic propagation velocity. Assume the history distance of the reference point (i.e., O point) is $R_{ref}$. Then the jamming signal of reference point can be expressed as below

(6) $$s_{J_{ref}}\left(\hat{t},t_{m}\right)=rect\left(\frac{\hat{t}-\frac{R_{ref}}{c}}{T_{p}}\right)\cdot \exp\left(j2\pi \left(f_{0}\left(t-\frac{R_{ref}}{c}\right)+\frac{\gamma }{2}{\left(\hat{t}-\frac{R_{ref}}{c}\right)}^{2}\right)\right)$$

Cast $p(\hat{t})$ into Equation (5) and de-chirp it with the reference signal, which reduces the bandwidth. Then the jamming signal can be expressed as

(7) $$\begin{array}{c} s_{J_{pf}}(\hat{t},t_{m})=\sigma_{i}rect(\frac{\hat{t}-\frac{R_{t}}{c}}{T_{p}})\cdot \exp(-\frac{j4\pi \gamma }{c}(t-\frac{2R_{ref}}{c})R_{\Delta })\\ \;\;\;\;\;\;\;\;\;\;\;\cdot \exp(-\frac{j4\pi \gamma }{c^{2}}R_{{}_{\Delta }}^{2})\exp(-\frac{j4\pi f_{0}R_{\Delta }}{c})\cdot rect(\frac{\hat{t}}{\tau })*\sum_{l_{r}=-\infty }^{+\infty }\delta \left(\hat{t}-l_{r}T_{r}\right) \end{array}$$

where

(8) $$\begin{array}{ll} R_{\Delta } & =\left\|R_{p}\right\|-\left\|R_{ref}\right\|\\ & =\left(\left\|R_{rj}\right\|+\left\|R_{OJ}\right\|+\left\|R_{Or}\right\|+2\left(x\cos\left(\omega t_{m}+\frac{\alpha }{2}\right)-y\sin\left(\omega t_{m}+\frac{\alpha }{2}\right)\right)\cos\frac{\alpha }{2}\right)\\ & -\left(\left\|R_{rj}\right\|+\left\|R_{OJ}\right\|+\left\|R_{Or}\right\|\right)\\ & =2\left(x\cos\left(\omega t_{m}+\frac{\alpha }{2}\right)-y\sin\left(\omega t_{m}+\frac{\alpha }{2}\right)\right)\cos\frac{\alpha }{2} \end{array}$$

When $\Delta \theta =\omega t_{m}$ is a slight angle, there is an approximation of Equation (8) as

(9) $$R_{\Delta }\approx 2\left(x\cos\frac{\alpha }{2}-\cos\frac{\alpha }{2}y\omega t_{m}+y\sin\frac{\alpha }{2}\right)\cos\frac{\alpha }{2}$$

Omitting the constant term and calculating the Fourier transform of $\hat{t}$ in Equation (7), then the ISRJ signal after range compression can be expressed as

(10) $$\begin{array}{c} s_{J_{pf}}\left(f,t_{m}\right)=\sum_{l_{r}=-\infty }^{+\infty }\sigma_{p}\tau f_{r}T_{r}\cdot \sin c(l_{r}\tau f_{r})\\ \;\;\;\;\;\;\;\;\;\;\;\sin c\left(T_{p}\left(f-\frac{\gamma R_{\Delta }}{c}-l_{r}f_{r}\right)\right)\cdot \exp\left(j2\pi \frac{f_{0}}{c}R_{\Delta }\right) \end{array}$$

Based on the frequency-distance relationship of de-chirping, $f=-\gamma \frac{2r}{c}$, the jamming signal is equivalent to

(11) $$\begin{array}{c} s_{J_{pf}}\left(r,t_{m}\right)=\sum_{l_{r}=-\infty }^{+\infty }\sigma_{p}f_{r}T_{r}\cdot \sin c(l_{r}\tau f_{r})\\ \;\;\;\;\;\;\;\;\;\;\;\;\sin c\left(\frac{4B}{c}\left(r+\frac{R_{\Delta }}{2}+\frac{l_{r}f_{r}c}{2\gamma }\right)\right)\cdot \exp\left(-j2\pi \frac{f_{0}}{c}R_{\Delta }\right) \end{array}$$

where r represents the range unit and B is the bandwidth of LFM signal. Actually, intermittent sampling in the fast time domain is equivalent to applying ISRJ jamming along the range orientation, and the ISAR image of false target can be obtained along the range orientation with ISRJ. Equation (11) illustrates that the jamming signal consists of a few sinc functions, which represent the strong scattering centers. This equation describes multiple peaks of the sinc function along the range orientation at $r=-R_{\Delta }/2-lf_{r}c/2\gamma$, which represents the results of the ISRJ signal with the function $p(\hat{t})$. Assuming the radar receives the target echo with the same parameters, the target echo signal can also be processed along the cross-range direction with an interrupted sampling repeater. 

The ISRJ signal in the azimuth dimension is similar with that in the range dimension that the jamming signal can be generated by interrupted transmitting the radar signal in the slow time domain. Multiplying $p(t_{m})$ by Equation (11), the jamming signal along two dimensions can be expressed as

(12) $$\begin{array}{ll} s_{J_{pf,a}}\left(r,t_{m}\right) & =p\left(t_{m}\right)\cdot s_{J_{pf}}\left(r,t_{m}\right)\\ & =rect(\frac{t_{m}}{\tau })*\sum_{l_{a}=-\infty }^{+\infty }\delta \left(t_{m}-l_{a}T_{a}\right)\cdot \sum_{l_{r}=-\infty }^{+\infty }\sigma_{p}f_{r}T_{p}\sin c\left(l_{r}\tau f_{r}\right)\\ & \cdot \sin c\left(\frac{4B}{c}\left(r+\frac{R_{\Delta }}{2}+\frac{l_{r}f_{r}c}{2\gamma }\right)\right)\cdot \exp\left(-j2\pi \frac{f_{0}}{c}R_{\Delta }\right) \end{array}$$

Casting Equations (9)–(12) and applying the Fourier transform in slow time, the jamming signal can be obtained. The distance variable along range orientation can be omitted when the variation of the Doppler Domain is discussed. The jamming signal can be expressed as

(13) $$\begin{array}{ll} s_{J_{pf,a}}\left(r,f_{m}\right) & =\sum_{l_{r}=-\infty }^{+\infty }\sigma_{p}f_{r}T_{p}\cdot \sin c\left(l_{r}\tau f_{r}\right)\cdot \sin c\left(\frac{4B}{c}\left(r+\frac{R_{\Delta }}{2}+\frac{l_{r}f_{r}c}{2\gamma }\right)\right)\\ & \cdot \sum_{l_{a}=-\infty }^{+\infty }\sin c\left(NT_{PRI}\left(f_{m}-\frac{2f_{0}}{c}\cos\frac{\alpha }{2}y\omega -l_{a}f_{a}\right)\right) \end{array}$$

where N is the number of pulse strings and $T_{PRI}$ is the pulse repetition interval. Equation (13) shows that peaks of the *sinc* function along the azimuth orientation are also located at $f_{m}=\frac{2f_{0}}{c}\cos\frac{\alpha }{2}y\omega +l_{a}f_{a}$ after pulse compression. 

According to ISAR principle, the ISAR image of the target is obtained along with the range orientation and azimuth orientation [27]. Thus, the ISRJ signal will induce a set of false-targets symmetrically centered on the real target along two dimensions of the RD image. The range interval of the false target is $f_{r}c/2\gamma$, and the azimuth interval of the false target is $f_{a}$. The number and amplitude of false-target are related to jamming sampling interval and duty cycle. Even though the number of targets on the RD image are increased after interrupted sampling repeater jamming, the false target on the RD can also be deemed to be sparse. 

## 3. The Theory of Adaptive CS-Based Method against ISRJ

According to the basic idea of the CS, the sparse signal can be reconstructed by a few measurements. A few scattered points of the target contained the most energy of target echo, thus the target echo signal is discrete and sparse. In that case, both the target echo and ISRJ signal can be reconstructed based on CS theory. In the following part, the ISAR imaging method based on CS theory is briefly introduced firstly. Then it is the CS reconstruction of the ISRJ signal. The last subsection describes and analyzes the anti-jamming procedure of the CS-based method.

### 3.1. ISAR Imaging via CS

The CS imaging of ISAR includes two steps. The first step is the measured process. The ISAR measured data y, which is accepted by the radar receiver, can be expressed as

(14) y=Φx+n

where $\Phi \in C^{M\times N}$ is the measurement matrix with M<N and $n\in C^{M}$ denotes the measured noise. Assume $x\in C^{N}$ represents the interested original signal. Generally, x can be represented as

(15) x=ΨΘ

where $\Psi \in C^{N\times N}$ is the orthonormal base matrix and Θ is a sparse weighting coefficients vector. If there are only K nonzero coefficients for Θ at most with K≪N, then the signal x is called owning K sparsity in the base matrix Ψ. Actually, x and Θ are two representations of the same signal that x is the complete signal representation, while Θ is the sparse representation of x in the sparse domain Ψ. Substituting Equations (15) to (14) and assuming that A is the dictionary matrix, which is represented as A=ΦΨ, we have

(16) y=AΘ+n

The second step is signal recovery. The sparse weighting coefficients vector can be obtained from limited measurement by calculating the convex optimization problem below

(17) $$\min\left({\left\|\Theta^{\prime }\right\|}_{p}\right),\;subject\;to\;{\left\|y-A\Theta^{\prime }\right\|}_{2}<\;\varepsilon$$

where ${\left\|\cdot \right\|}_{p}$ stands for $l_{p}$ norm and min(⋅) represents the minimization, $\Theta^{\prime }$ represents the coefficient vector to calculate, and ε is the noise level. Equation (16) will have a solution in the condition of matrix A obeying the RIP condition [20]. Then the ISAR image can be obtained by reconstructing pulse signal firstly and range unit signal secondly based on CS. 

### 3.2. ISRJ Signal Imaging via CS 

The ISRJ signal can be deemed sparse because the multiple false-targets consist of a few strong scattering centers in ISAR imaging. Thus, the ISRJ jamming signal can be collected and recovered with CS theory. This section analyzes the CS-based anti-jamming model of 2D ISRJ jamming signal. The anti-jamming process is divided into two the 2D CS reconstruction. 

The first step is countering the range orientation jamming by reconstructing the jamming signal with the CS method. The down-range orientation of the ISRJ signal is applied with de-chirping and Fourier transform, then the ISRJ signal model with 2D interrupted sampling can be simplified from Equation (15) as

(18) $$\begin{array}{ll} s_{J_{pf}}\left(r,t_{m}\right) & =\sum_{p=1}^{P}\sum_{l_{r}=-\infty }^{+\infty }a_{p}^{*}\cdot \sin c(l_{r}\tau f_{r})\delta \left(\frac{4B}{c}\left(r+\frac{R_{\Delta }}{2}+\frac{l_{r}f_{r}c}{2\gamma }\right)\right)\\ & \cdot rect(\frac{t_{m}}{\tau })*\sum_{l_{a}=-\infty }^{+\infty }\delta \left(t_{m}-l_{a}T_{a}\right) \end{array}$$

where $a_{p}^{*}=\sigma_{p}f_{r}T_{r}\cdot \exp\left(j2\pi \frac{f_{0}}{c}R_{\Delta }\right)$. Assuming that the observation region along range orientation is $\left[r_{0},r_{1}\right]$, the $\rho_{r}$ represents the range resolution, and the number of bins along range orientation is $N=\frac{r_{1}-r_{0}}{\rho_{r}}$. In the ISAR image domain, the total number of scatters is P. Then the observation matrix can be constructed as

(19) $$\begin{array}{c} A={\left[\varphi_{1},\ldots ,\varphi_{n},\ldots ,\varphi_{N}\right]}_{1\times N}\\ \varphi_{n}=\sum_{l_{r}=-\infty }^{+\infty }\delta \left(\frac{4B}{c}\left(r+\frac{\left(r_{0}+n\rho_{r}\right)}{2}+\frac{l_{r}f_{r}c}{2\gamma }\right)\right) \end{array}$$

Suppose the number of bins along azimuth orientation is I, then the dimension of the matrix A is I×N. The scattering echo coefficient vector of one pulse is

(20) $$\begin{array}{c} \Theta^{T}={\left[\Theta_{1},\ldots ,\Theta_{n},\ldots ,\Theta_{N}\right]}_{1\times N}\\ \Theta_{n}=\sum_{l_{a}=-\infty }^{+\infty }\sum_{n=1}^{N}\sigma_{n}f_{r}T_{r}\exp\left(j2\pi \frac{f_{0}}{c}\left(r_{0}+n\rho_{r}\right)\right)rect(\frac{t_{m}}{\tau })*\sum_{l_{a}=-\infty }^{+\infty }\delta \left(t_{m}-l_{a}T_{a}\right) \end{array}$$

where $\sigma_{n}$ represents the n-th backscattering coefficient along the range orientation. Moreover, $\sigma_{n}=0$ means there is no target in the range units. If the false targets occupy only a small part of the observation area, the number of nonzero elements in Θ is much smaller than the number of distance units N. The de-chirping echo signal of the jamming signal can be expressed as

(21) $$s_{J_{pf}}=A\Theta +n$$

where the n is the additive noise and Θ is k-sparse vector, which can be estimated by calculating the convex optimization problem below

(22) $$\min\left({\left\|\Theta^{\prime }\right\|}_{p}\right),\;\mathrm{subject}\;\mathrm{to}\;{\left\|s_{J_{pf}}-A\Theta^{\prime }\right\|}_{2}<\;\varepsilon$$

where $s_{J_{pf}}$ represents one pulse echo signal.

The second step is the ISAR azimuth processing of the ISRJ signal based on CS. After range alignment and phase compensation, the ISRJ signal in one range unit after compression can be expressed as

(23) $$\begin{array}{c} s_{J\prime_{pf}}\left(t_{m}\right)=\sum_{i=1}^{I}\sum_{l_{a}=-\infty }^{+\infty }\sigma_{i}f_{r}T_{p}\exp\left(j2\pi \frac{f_{0}}{c}R_{\Delta }\right)\\ \;rect(\frac{t_{m}}{\tau })*\sum_{l_{a}=-\infty }^{+\infty }\delta \left(t_{m}-l_{a}T_{a}\right) \end{array}$$

where $\sigma_{i}$ represents the i-th backscattering coefficient along with the azimuth orientation. The reflection coefficient of the background is zero. Substituting Equation (9) into Equation (23) and ignoring the RVP, the jamming signal model of ISAR image in one range unit after compression can be represented as

(24) $$s_{J\prime_{pf}}\left(f_{m}\right)=\sum_{i=1}^{I}\sum_{l_{a}=-\infty }^{+\infty }\sigma_{i}f_{r}T_{p}\cdot \sin c\left(NT_{PRI}\left(f_{m}-\frac{2f_{0}}{c}\cos\frac{\alpha }{2}y\omega -l_{a}f_{a}\right)\right)$$

Assuming that the observation region along azimuth orientation is $\left[y_{0},y_{1}\right]$, the $\rho_{a}$ represents the azimuth resolution, and the number of bins along range orientation is $N=\frac{y_{1}-y_{0}}{\rho_{a}}$. Then the observation matrix of jamming signal in one range unit can be described as

(25) $$\begin{array}{c} \Psi^{T}={\left[\psi_{1},\ldots ,\psi_{i},\ldots ,\psi_{I}\right]}_{1\times I}\\ \psi_{i}=f_{r}T_{p}\delta \left(NT_{PRI}\left(f_{m}-\frac{2f_{0}}{c}\cos\frac{\alpha }{2}\left(y_{0}+n\rho_{a}\right)\omega -l_{a}f_{a}\right)\right) \end{array}$$

where Ψ is the observation matrix with I×N, which is different from the matrix A. Hence, the vectors of scatters along azimuth dimension can be obtained by calculating the optimization problem below.

(26) $$\min\left({\left\|\Theta_{a}\right\|}_{p}\right),\;\mathrm{subject}\;\mathrm{to}\;{\left\|s_{J\prime_{pf}}-\Psi \Theta_{a}\right\|}_{2}<\;\varepsilon$$

### 3.3. Anti-Jamming Procedure on CS Theory

According to the theory of CS-based ISAR imaging, the sparsity actually represents the scattered number of targets. Hence, the target can be reconstructed with the optimum sparsity on the condition of RIP, which is

(27) M≥cKlog(Q/K)

where M is the number of the measurements, K is the sparsity of vector, and c is a small constant. Q represents the dimension of the coefficient vector, which equals the number of distance units N and I respectively in Section 3.2. Assuming the minimum number of measurements satisfies the equation $M_{l}=cK\log(N/K)$, the signal can be reconstructed as long as the measurements are bigger than $M_{l}$. The relationship between sparsity and measurements number is proved and simulated in [22]

1. The probability of reconstruction increases with adding the measurements under invariant sparsity;
2. With the increasing sparsity, the reconstruction is guaranteed only by more measurements.

This conclusion is significant for suppressing the ISRJ signal below.

**Figure 2:**
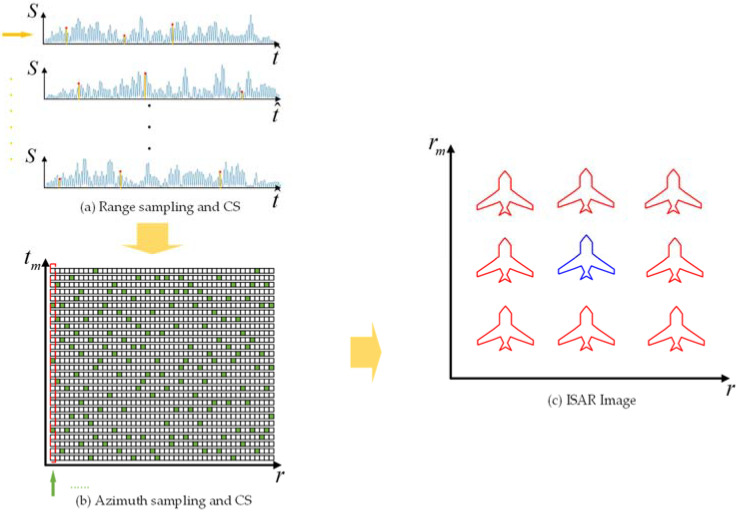
The CS-based anti-jamming method against ISRJ.

Section 2 describes that the ISRJ signal is generated by periodically sampling and forwarding the original target signal. Therefore, the ISRJ signal can induce multiple false targets with high similarity in the frequency domain. Figure 2 shows the CS-based anti-jamming method against ISRJ, and those characteristics of the ISRJ signal are revealed in it. The ISAR imaging process of the ISRJ signal is divided into two steps. 

Firstly, $M_{d}$ points are randomly sampled along with the range orientation for each echo signal, that is, along the direction of the yellow arrow in Figure 2a. Those red points represent the value that is sampled. Each echo’s high-resolution range profile (HRRP) can be obtained by reconstructing the vector based on the proposed CS method. 

Next, different reconstructed echo values in the range unit are randomly sampled $M_{a}$ points along the azimuth direction, which is the direction of the green arrow in Figure 2b. Those green blocks indicate the point being sampled. Then every column vector is reconstructed by the proposed CS method, and the imaging results of the ISRJ signal can be obtained. 

Figure 2c shows the dense false targets induced by the ISRJ signal, in which the blue aircraft represents the true target, and the red aircraft represents the false targets. Multiple false targets with high similarity are distributed as an equal interval. In [4], the main false target was in the middle of all false targets, and the amplitude of the main false target was stronger than that of other false targets. Besides, the main false target overlaps with the true target. These characteristics of ISRJ can be utilized for anti-jamming. The proposed CS method obtains K-sparse optimal solutions, which can express the most energy of the observation vector by solving optimization problems. Since the ISRJ signal is sparse in ISAR image domain, it can be reconstructed by CS method only with a few random measurements.

Figure 2 exhibits the scattering number of the ISRJ signal is more than those of the target signal without jamming. Hence, the sparsity of jamming signal $K_{J}$ is bigger than that of target signal K. Then the condition of reconstructing jamming signal can be represented from Equation (20)

(28) $$M_{J}\geq cK_{J}\log(N/K_{J})$$

**Figure 3:**
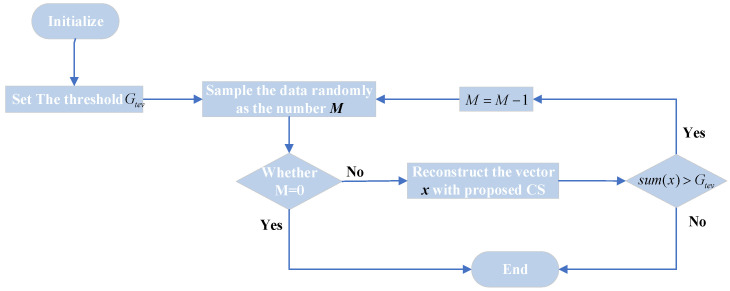
Flow chart of the anti-jamming method based on CS theory.

Let $M_{l}{}_{J}=cK_{J}\log(N/K_{J})$ indicate the minimum measurements needed to reconstruct the jamming signal, then $M_{l}{}_{J}>M_{l}$. There is a minimum anti-sampling number $M_{anti}$, which satisfies the equality $M_{l}<M_{anti}<M_{l}{}_{J}$. The amplitude of multiple false targets induced by the ISRJ signal is different. The energy of the main false target in the middle is the largest, and the energy of other false targets decreases in turn. Furthermore, the main false target overlaps with the real target. Therefore, the jamming signal can be suppressed with the number of the optimal measurements $M_{anti}$, by which the target can be reconstructed while the jamming signal cannot be reconstructed. Moreover, the false middle target and real target are reconstructed firstly with the largest energy because the energy is the largest. The one-dimensional procedure of the algorithm is shown in Figure 3. The 2-D imagery repeats this algorithm along with range and azimuth orientation. 

In this flowchart, parameters of system are initialized firstly, and a threshold $G_{tev}$ is set up. $G_{tev}$ is an empirical constant calculated from a distance between the target and the radar and the transmitting power of the jammer. Secondly, the number of measurements is calculated from M=cKlog(N/K), which can ensure complete reconstruction of the vector x. Thirdly, the vector x is reconstructed by the proposed CS method. It can be judged whether the ISRJ signal has been removed by the inequality $sum(x)>G_{tev}$. The overlapping of the main false targets and the true target will be reconstructed, which is mentioned earlier. Therefore, the energy of the jamming signal can be filtered by adjusting the number of measurements M. The energy of the reconstructed signal also includes the energy of the ISRJ signal when $sum(x)>G_{tev}$. Thus, the energy of sampled signal should be reduced by reducing the number of measurements (i.e., M=M−1). Until $sum(x)\leq G_{tev}$, the overlapping of true target and main false target will be reconstructed. 

## 4. Simulations

**Figure 4:**
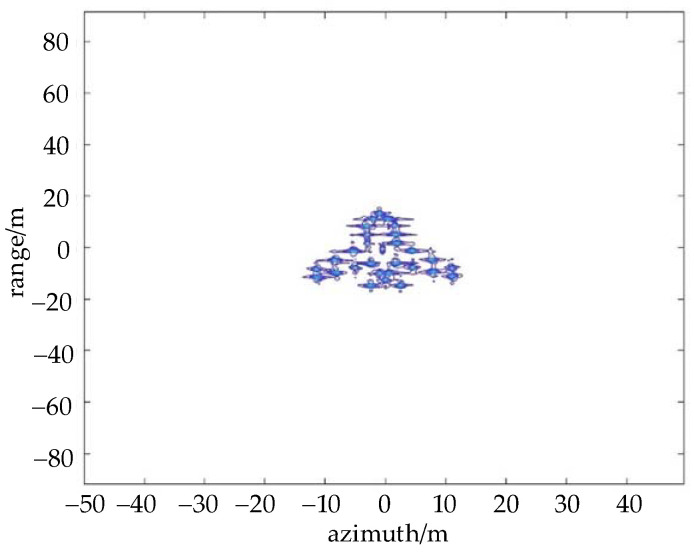
The ISAR image without ISRJ.

In this section, the performance of the anti-jamming method is validated by simulations. The main simulation parameters are listed in Table 1. Figure 4 shows the target in the ISAR image without the ISRJ signal.

### 4.1. Suppression Effect of ISRJ without Noise

There is usually a jamming signal in the magnetic environment. Table 2 lists the main jamming simulation parameters with a jamming-to-signal ratio (JSR) of 0 dB. The duty ratio of the ISRJ signal is D=0.67. The sampling frequency of ISRJ along with the range and azimuth orientation are $f_{r}$ and $f_{a}$, respectively. 

**Figure 5:**
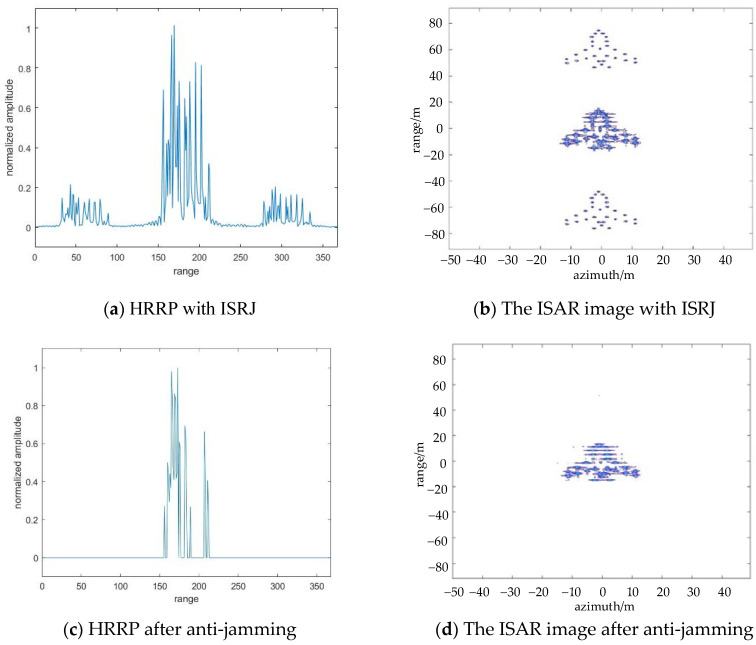
The results of ISRJ jamming and anti-jamming in the fast time domain, when $M_{d}=29,\;M_{a}=32$.

In this section, the suppression effect of ISRJ is analyzed without noise. Assume $M_{d}$ represents the sampling points of the received signal for CS theory that along the range direction, $M_{a}$ represents the sampling points number of the received signal along the azimuth direction for CS theory. According to the empirical threshold in the flowchart in Figure 3, the optimal numbers of anti-jamming measurements in different directions are separately obtained, $M_{d}=29,\;M_{a}=32$. Figure 5a,b show the HRRP and ISAR imaging results with ISRJ in the fast time domain. Under the optimal number of measurements, the CS method is used to process the received signal in the range direction. Figure 5c,d shows the anti-jamming results. It can be seen from Figure 5 that if the jammer only adopts under-sampled interference in the fast time domain, the jamming signal can be effectively suppressed on CS theory, which proves the suppression effect of under-sampled interference on the CS theory.

**Figure 6:**
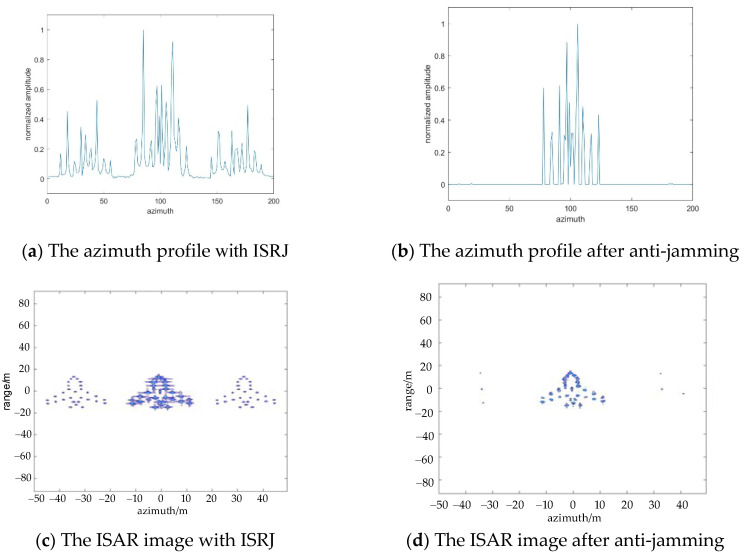
The results of ISRJ jamming and anti-jamming in the slow time domain, when $M_{d}=58,\;M_{a}=8$.

Next, the anti-interference of the algorithm against azimuth ISRJ interference is simulated. Figure 6a,c shows the azimuth profile and ISAR image with ISRJ in the slow time domain. Figure 6b,d are the results after anti-jamming. At this time, the calculated number of measurements in the range direction and azimuth direction is $M_{d}=58,\;M_{a}=8$. It is effectively suppressed the ISRJ signal that only the middle target is retained and false targets next to the middle targets are removed.

**Figure 7:**
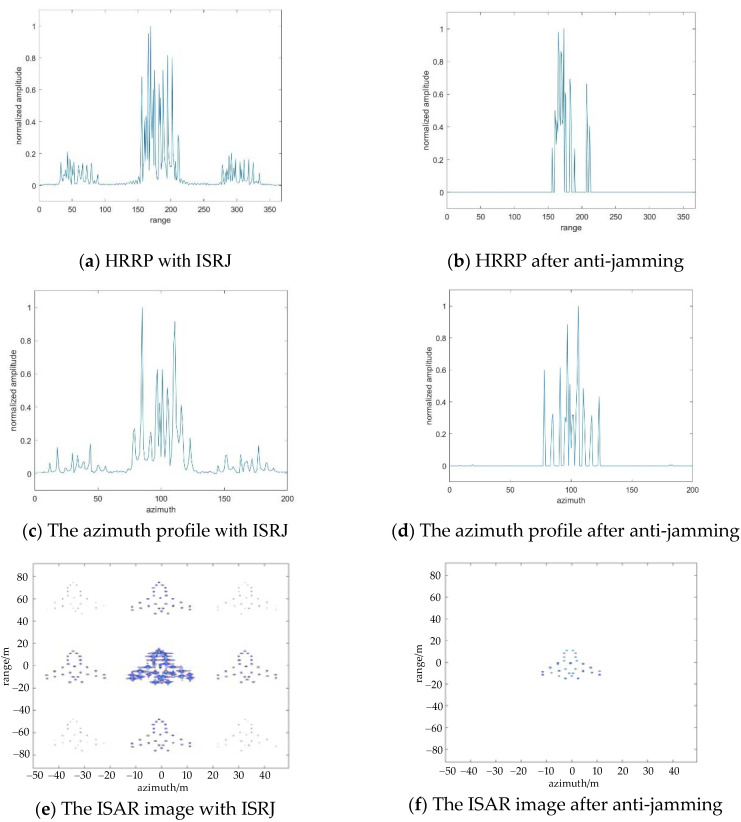
The results of ISRJ jamming and anti-jamming in the fast time domain, slow time domain, and ISAR image when $M_{d}=16,\;M_{a}=4$.

Figure 7 shows the anti-jamming process both in the slow time domain and fast time domain. Figure 7b,d,f show the HRRP, the azimuth profile, and the ISAR image after processing by the proposed method. At this time, the calculated number of measurements in the range direction and azimuth direction is $M_{d}=29,\;M_{a}=8$. In Figure 7e, multiple false targets are generated on the RD plane along with range orientation and azimuth orientation, and the main middle target is actually the overlap of the real target, and the primary false target and its power spectral density is larger than that of other secondary false targets. Hence, this number of measurements can make sure reconstruction of the main middle target, while the other false targets cannot be reconstructed. 

### 4.2. Suppression Effect of ISRJ under Different JSR

**Figure 8:**
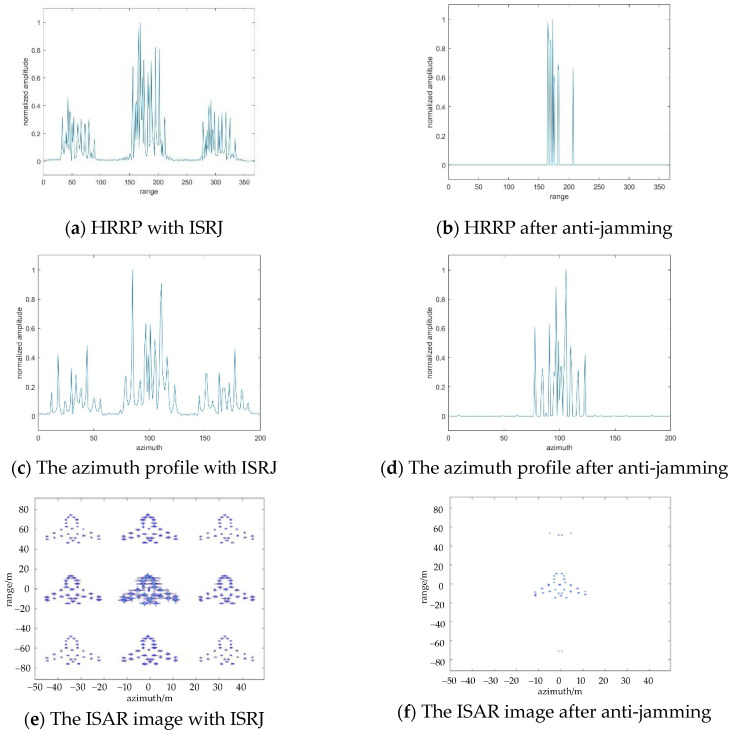
The results of ISRJ jamming and anti-jamming in the fast time domain, slow time domain, and ISAR image with JSR=20dB.

In the actual radar monitoring environment, the jamming power released by the jammer is usually greater than the radar’s original signal power. JSR has an important influence on the jamming effect. The change of anti-jamming effect with JSR will be analyzed in the following part. Figure 8 shows the anti-jamming comparison when JSR=20dB. At this time, the calculated number of measurements in the range direction and azimuth direction is $M_{d}=16,\;M_{a}=4$. Figure 8b,d,f show the anti-jamming results of HRRP, azimuth orientation, and ISAR image, respectively. Even though the power of the jamming signal is much higher than the power of the real target echo signal, the anti-jamming effect is good that the false target can still be suppressed. 

**Figure 9:**
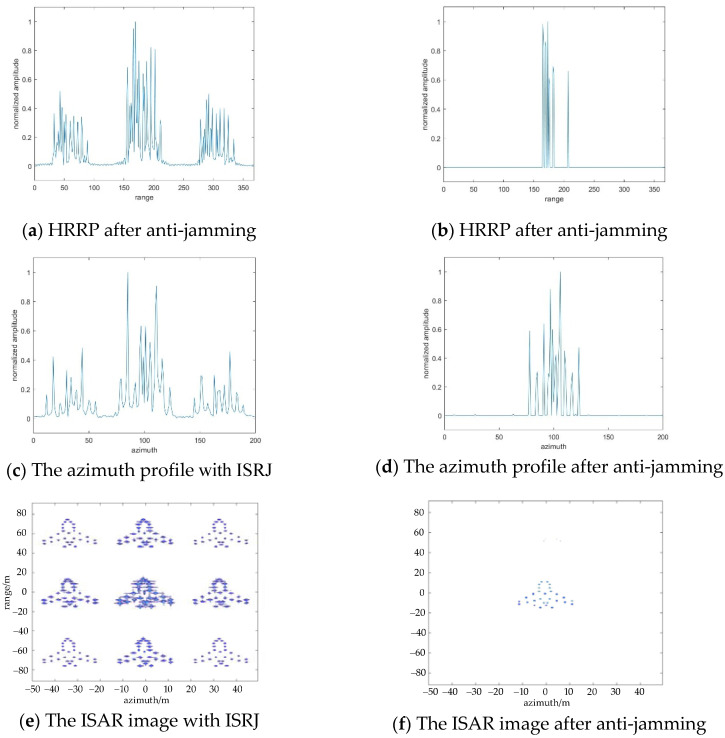
The results of ISRJ jamming and anti-jamming in the fast time domain, slow time domain, and ISAR image with JSR=40dB.

Figure 9 shows the anti-jamming result when JSR=40dB. By comparing Figure 7, Figure 8 and Figure 9 e,f, it is verified that the proposed CS method still has good anti-jamming performance even when the ISRJ signal power is set up much greater.

### 4.3. Suppression Effect of ISRJ under Different SNR

**Figure 10:**
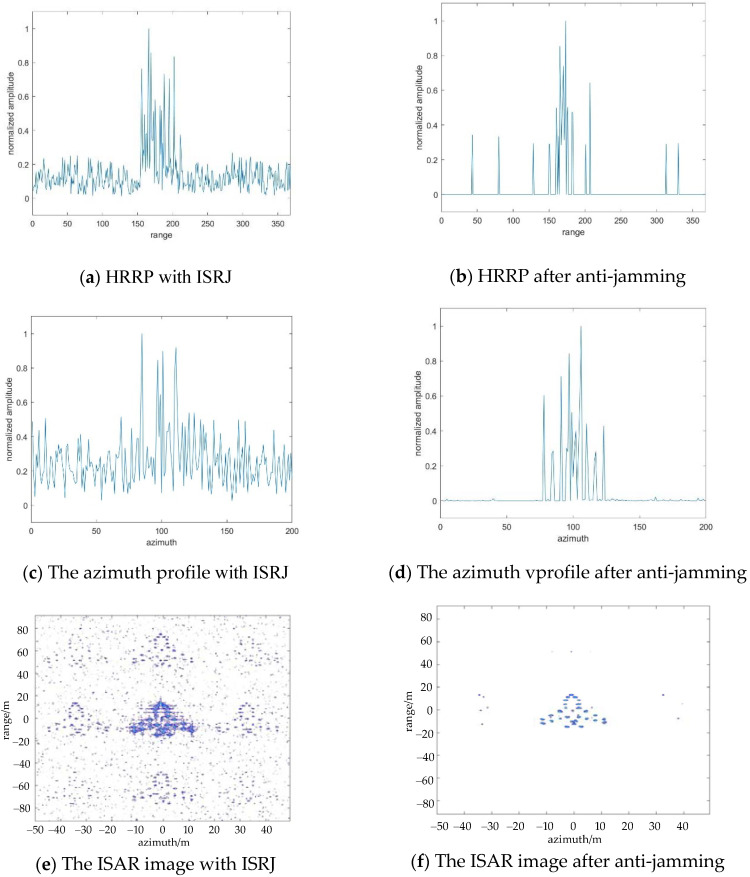
The results of ISRJ jamming and anti-jamming in the fast and slow time domain with JSR=0dB and SNR=-5dB.

Both Gaussian white noise and man-made jamming noise exist in the actual electromagnetic environment. In order to verify the anti-jamming effect of the proposed method in the actual situation, this part conducts a simulation of the ISRJ suppression under different combinations of SNR and JSR. Figure 10 shows the anti-jamming results with JSR=0dB and SNR=-5dB. According to Figure 10e, when the Gaussian white noise is taken into account in the simulation, spots on the imaging plane will seriously affect the imaging quality. The power of the ISRJ interference signal is small, and it is almost submerged by Gaussian white noise. Figure 10b,d, respectively, show the HRRP and the azimuth profile of received signals, processed sparsely by the proposed method. At this time, the calculated number of measurements is $M_{d}=16,\;M_{a}=4$. The spectrum of Gaussian noise is evenly distributed on the ISRJ signal and real target signal, thus it will not affect the anti-jamming effect of the proposed method. Then it can effectively suppress false targets in the range direction and azimuth direction by processing signals with CS theory in the fast-time domain and slow-time domain, respectively. 

**Figure 11:**
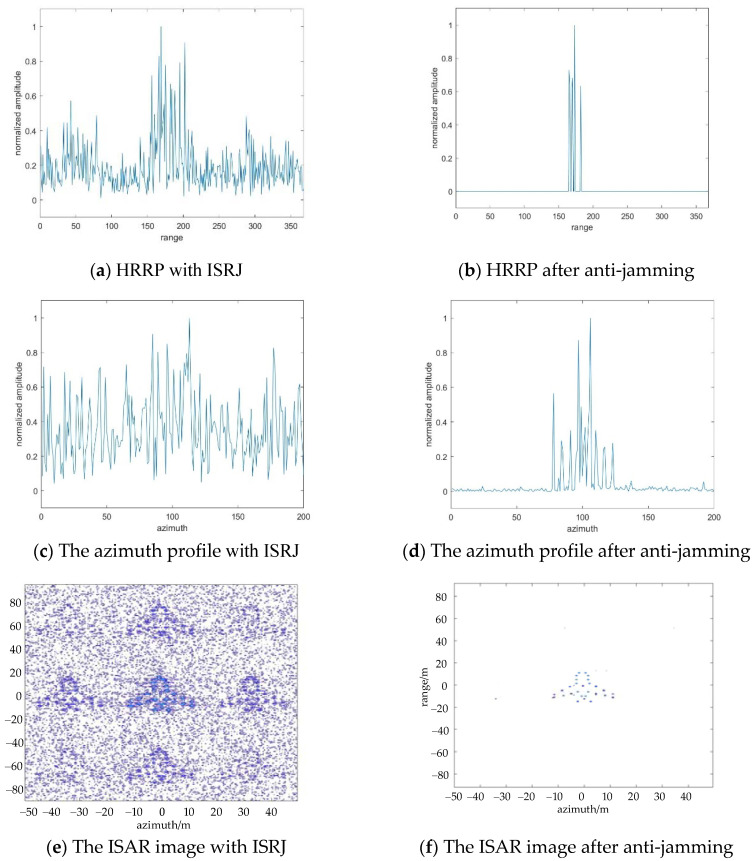
The results of ISRJ jamming and anti-jamming in the fast and slow time domain with JSR=20dB and SNR=-20dB.

When ISRJ signal power and noise power are larger, let us observe the anti-jamming performance of the method. Figure 11 shows the ISRJ effects and anti-jamming effects with JSR=20dB and SNR=-20dB. Figure 7a is the HRRP image with under-sampled interference in the noisy environment. Figure 7b is the ISAR image with under-sampled interference. Compared to Figure 6b and Figure 7b, it can be seen that with the decrease of SNR, the imaging effect gradually deteriorates. Figure 7c,d, respectively, show the HRRP and ISAR image after distance and azimuth processing with CS theory. At this time, the calculated number of measurements is $M_{d}=10,\;M_{a}=4$. Figure 7d shows that under the premise of imaging the real target effectively, CS theory can still achieve effective suppression of interference signals even if the noise power is increased. Comparing Figure 9 and Figure 10, shows that the method can also suppress the Gaussian white noise in the anti-jamming process.

### 4.4. Suppression Effect of ISRJ under Measured Data

**Figure 12:**
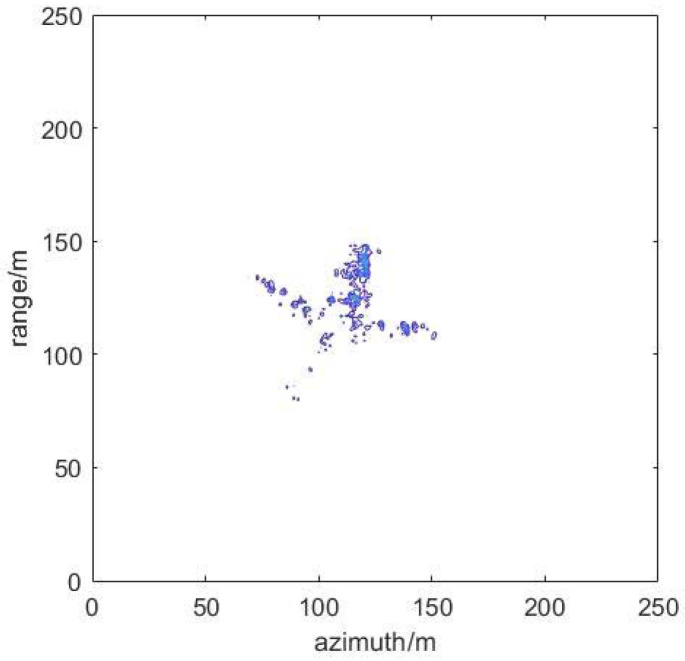
ISAR imaging of Yak-42.

**Figure 13:**
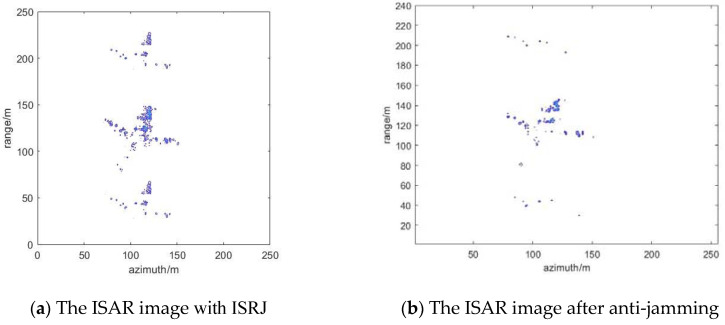
The results of ISRJ jamming and anti-jamming in the fast time domain.

**Figure 14:**
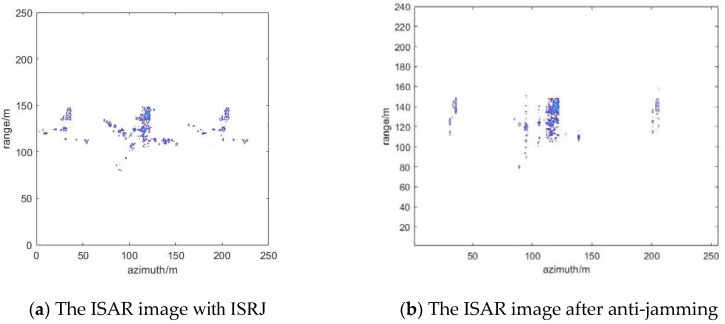
The results of ISRJ jamming and anti-jamming in the slow time domain.

**Figure 15:**
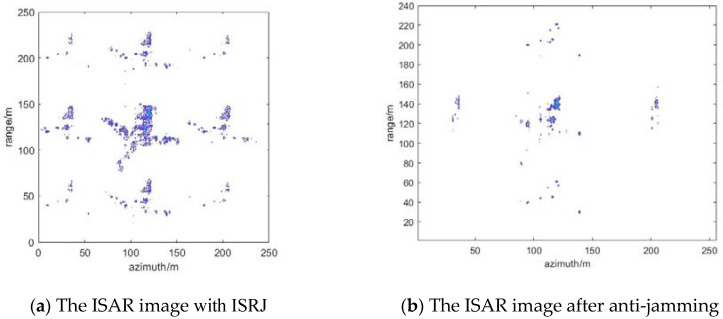
The results of ISRJ jamming and anti-jamming both in the fast time domain and in the slow time domain.

The published Yak-42 aircraft measured data are used to verify the method below. Meanwhile, the parameters of the ISRJ signal are further improved. Figure 12 shows ISAR imaging of Yak-42. Figure 13 shows the results of ISRJ jamming and anti-jamming in the fast time domain. The anti-jamming measurement number is obtained by the proposed method, M_ d = 14, M_ a = 22. Figure 14 shows the results of ISRJ jamming and anti-jamming in the slow time domain. Moreover, the measurement number is M_ d = 22, M_ a = 7. Figure 15 shows the results of ISRJ jamming and anti-jamming both in the fast time domain and in the slow time domain. Moreover, the measurement number is M_ d = 14, M_ a = 12.

From Figure 13b, Figure 14b and Figure 15b, it can be observed that the real target in the middle of the anti-jamming ISAR image is not clear. This is because the number of strong scatters of the real target is less than that of the ideal model, and the imaging of Yak-42 is not very clear without the ISRJ jamming. Then, the target echo signal energy will be weaker after anti-jamming. Hence, the imaging quality of the target is not very good after anti-jamming, while the target contour can still be distinguished. In addition, some noise points exist in the anti-jamming ISAR image along the range direction and azimuth direction, respectively, and its anti-jamming effect is not as good as that in the previous three sections. Nevertheless, the distinguished contour of the target can verify the effectiveness of the proposed anti-jamming method in this paper.

## 5. Conclusions

This paper studied the anti-jamming method of ISAR imaging based on CS theory. The effects of SNR, sampling points of received signals, and JSR on anti-interference are analyzed, respectively. Simulation results show that the proposed method can effectively suppress the ISRJ signal. In this method, reducing the measurements number of echo signals can decrease the sampling probability of sub-false targets. The main middle target is the overlap of true targets and main false targets. Thus, the measurements can reconstruct the main middle target, which owns larger power, and the other false targets will be suppressed. Moreover, the anti-jamming performance of this method will not weaken with the increase of JSR. The biggest challenge of the method proposed in this paper is that the anti-jamming effect depends on the threshold. Whether the threshold is appropriate or not determines the anti-jamming effect. Bayesian compressed sensing can be a potential direction to generate appropriate threshold adaptively in the future.

## Figures and Tables

**Table 1:** Simulation parameters.

Parameters	Numerical Value	Parameters	Numerical Value
$f_{0}\left(\mathrm{GHz}\right)$	10	PRF(Hz)	200
B(MHz)	300	ω(rad)	0.02
$T_{p}\left(us\right)$	1	α(rad)	0

**Table 2:** Jamming simulation parameters.

Parameters	Numerical Value	Parameters	Numerical Value
$f_{r}\left(\mathrm{MHz}\right)$	50	$f_{a}\left(\mathrm{Hz}\right)$	66.67
